# Methanolic Crude Extract of *Hagenia abyssinica* Possesses Significant Antidiarrheal Effect: Evidence for *In Vivo* Antidiarrheal Activity

**DOI:** 10.1155/2021/9944629

**Published:** 2021-05-16

**Authors:** Zemene Demelash Kifle, Seyfe Asrade Atnafie, Tesfaye Yimer Tadesse, Teshome Fentik Belachew, Birhanu Berihun Kidanu

**Affiliations:** ^1^University of Gondar, College of Medicine and Health Sciences, School of Pharmacy, Department of Pharmacology, Gondar, Ethiopia; ^2^Debretabor University, College of Health Science, Department of Pharmacy, Debre Tabor, Ethiopia; ^3^Department of Pharmacy, Debre Birhan Health Science College, Debre Birhan, Ethiopia; ^4^University of Gondar, College of Veterinary Medicine and Animal Sciences, Department of Veterinary Pharmacy, Gondar, Ethiopia

## Abstract

**Background:**

*Hagenia abyssinica* is one of the most commonly used medicinal plants for the treatment of diarrhea in Ethiopia. Therefore, this study aimed to evaluate the antidiarrheal effect of methanol crude extract of *H. abyssinica* leaves in mice.

**Methods:**

Acute toxicity testing was conducted using Organization for Economic Cooperation and Development guidelines. The antidiarrheal activity of the crude extract of *H. abyssinica* was investigated using three animal models such as small intestine transit, enteropooling, and castor oil-induced diarrhea models. The extract was administered at three different doses (100, 200, and 400 mg/kg) to the test groups, while the positive control group received 3 mg/kg of loperamide and the negative control group received 10 ml/kg of vehicle (distilled water).

**Results:**

The crude extract of *H. abyssinica* did not exhibit death at the limit dose (2 g/kg) throughout the observation period. In the castor oil-induced model, the crude extract at a dose of 200 and 400 mg/kg exhibited a significant (*P* < 0.05) antimotility effect as compared to the negative control. The crude extract revealed a significant reduction in the volume and weight of intestinal contents at 200 and 400 mg/kg doses of the extract. Moreover, the highest antidiarrheal index (ADI) was obtained with the dose of 400 mg/kg of crude extract, which was comparable to the standard drug.

**Conclusion:**

The crude extract of *Hagenia abyssinica* possesses antidiarrheal activity and supports the traditional use of *H. abyssinica* for the management of diarrhea.

## 1. Introduction

According to the World Health Organization, diarrhea is the passage of three or more loose or liquid stools per day [1]. Although diarrhea is a preventable and treatable disease, it is the 2^nd^ leading cause of death in children under 5 years old [2]. Globally, there are nearly 1.7 billion cases of childhood diarrheal disease every year. Diarrhea is a leading cause of malnutrition in children under five years old and kills around 525, 000 children under five [1].

Diarrhea is the most common problem in low-income countries like Africa. In Africa, about 26% of incidents of diarrhea were reported [3]. Patients with mild diarrhea have a self-limited sickness necessitating no management. However, in severe diarrhea, risks like electrolyte imbalance and dehydration are common, predominantly in elderly, children, and infant patients. These patients require zinc supplements, antimicrobials, oral rehydration therapy, antisecretory agents, and antimotility agents [4].

The World Health Organization estimated that about 80% of the population in low-income countries depend on plant-based medicines [5]. Numerous herbal medicines that possess antidiarrheal effects are existing in the biosphere. The antidiarrheal effects of these herbal medicines have been accredited to the presence of phytoconstituents such as tannins, flavonoids, alkaloids, steroids, terpenoids, and saponins [6]. In the previous study, the crude leaf extract of *H. abyssinica* comprises phytoconstituents such as flavonoids, phenols, anthraquinones, triterpenoids, and saponins [7]. Similarly, the leaf solvent fractions of *H. abyssinica* contain secondary metabolites such as alkaloids, flavonoids, tannins, glycosides, phenols, anthraquinones, terpenoids, and saponins [8].

Scientific investigation for the search on novel antidiarrheal compounds from medicinal plants such as *Caylusea abyssinica* [9], *Ixora coccinea* [10], *Ficus sycomorus* [11], *Vernonia amygdalina* [12], *Myrtus communis* [13], and *Justicia schimperiana* [14] revealed promising findings. The medicinal plant *H. abyssinica* belongs to the genus Hagenia and the family Rosaceae [15]. The species occurs in Tanzania, Kenya, Uganda, Congo, Ethiopia, Malawi, Burundi, Rwanda, and Sudan [16]. The whole plant is traditionally used for the treatment of fever/cough, stomachache, livestock disease, and malaria treatment. The flower part of *H. abyssinica* is used for tapeworm, epilepsy, wound healing, evil eye, problems related to bile, and sexually transmitted diseases [17, 18]. The leaf of *H. abyssinica* has been used in the treatment of diarrhea in Ethiopian folk medicine [17, 19, 20]. Pharmacological investigation reports showed that the extract of *H. abyssinica* has a considerable antibacterial activity against the bacterial species (*Staphylococcus aureus* and *Salmonella typhi*) [21]. In the previous study, the leaf solvent fractions of *H. abyssinica* showed significant antidiarrheal activity in mice [8]. However, there is no previous study in the *in vivo* antidiarrheal effect of the leaf crude extract of *H. abyssinica* in Swiss albino mice. Thus, the present study aimed to investigate the *in vivo* antidiarrheal effect of the leaf crude extract in Swiss albino mice.

## 2. Materials and Methods

### 2.1. Drugs, Chemicals, and Instruments

Loperamide hydrochloride (Brawn Laboratories Ltd., India), activated charcoal (Acuro Organics Ltd., New Delhi), methanol (Blulux, India), castor oil (Amman Pharmaceutical Industries, Jordan), hot air oven (Medite Medizintechnik, Germany), vacuum freeze dryer (Labfreez Instruments Group Co., Ltd., Germany), digital thermometer (Infiniti Medlab Pvt., Ltd., India), and microhematocrit centrifuge (Medite Medizintechnik, Germany) were used in the study.

### 2.2. Plant Materials

The fresh leaves of *H. abyssinica* were collected in Kosoye, Ethiopia, on February 12, 2019, and wrapped with plastic sheets during transportation. The botanical identification and authentication of the plant material were performed by a botanist at the University of Gondar, Ethiopia, with the voucher specimens' number 003ZDK/2019.

### 2.3. Extraction

The collected plant leaves were shade-dried at room temperature. The powdered plant materials were weighed by sensitive digital weighing balance, and a total of 1050 g of powdered leaves were macerated with 80% methanol (262.5 g in 1500 ml) in four Erlenmeyer flasks for 72 h at room temperature with occasional stirring and shaking, and then, the extracts were filtered by using Whatman filter paper No.1. The marc was remacerated two times with fresh solvent, each for 72 h, and the filtrates obtained from the successive maceration were concentrated in a rotary evaporator (Hamato, Japan) under reduced pressure at 40°C. The semidried residues were frozen in the refrigerator overnight and then dried using a lyophilizer (Labfreez, China) to completely remove the solvent residue. Then, the dried leaves' extract was kept separately in a desiccator until used for the experiment [22, 23].

### 2.4. Experimental Animal Handling, Grouping, and Dosing

Adult Swiss albino mice of either sex weighing between 25 and 30 g were purchased. The animals were housed in a standard polyethylene glycol cage, with the provision of a standard diet and water ad libitum. The animals were allowed for a one-week acclimatization period. These animals were kept under standard conditions of humidity, temperature, and 12 h light/12 h dark cycle. During the experimental period, the animals were randomly assigned into five groups (negative control, three test groups, and positive control) comprising six animals per group. Group I (negative controls) were treated with vehicle (distilled water); group II, III, and IV were treated with 100, 200, and 400 mg/kg of the crude extract of *H. abyssinica*, respectively, while group V received loperamide (3 mg/kg) orally.

### 2.5. Acute Toxicity Testing

Acute toxicity testing was conducted using Organization for Economic Cooperation and Development guidelines. Accordingly, five female albino mice of 8–12 weeks were used. All mice were fasted (food but not water) overnight before and 2 h after the administration of the extract. The first animal was given a limit dose of 2 g/kg, and then four other mice were sequentially treated based on the outcome of the first animal and observed for any signs of toxicity daily for 14 days such as loss of appetite, hair erection, lacrimation, tremors, convulsions, salivation, diarrhea, mortality, and other signs of toxicity [24].

### 2.6. Castor Oil-Induced Diarrhea Model

The method used was described by Kifle and Molla [8, 25], with a slight modification. Thirty Swiss albino mice of either sex were fasted for about 18 h and grouped randomly into 5 groups as mentioned above. After 1 h of treatment with the vehicle, 100, 200, and 400 mg/kg of the crude extract, and loperamide hydrochloride (3 mg/kg), diarrhea was induced by oral administration of 0.5 ml of castor oil to each mouse. Then, the mice were placed individually into metabolic cages in which the floor is lined with transparent paper for the collection of fecal matters. The transparent paper altered every one hour for a total of four hours. The mice were observed for a period of four hours. The mice were removed from their cages, and the weight of feces was obtained by subtracting the weight of filter paper from the weight of feces and filter paper. The number of wet stools, the onset of diarrhea, the total number, and the total weight of fecal output were noted. The percentage of the weight of total fecal output and the percentage of diarrheal inhibition were determined by using the following formulas:

(1) $$\begin{array}{c} \text{percentage inhibition}\left(\%\right)=\frac{\text{mean number of WFC}-\text{mean number of WFT}\times 100}{\text{mean number of WFC}}, \end{array}$$

where WFC is the wet feces in the control group and WFT is the wet feces in the test group, and

(2) $$\begin{array}{c} \text{percentage of fecal output}\left(\%\right)=\frac{\text{mean fecal weight of each treatment group}\times 100}{\text{mean fecal weight of control}}. \end{array}$$

### 2.7. Castor Oil-Induced Intestinal Transit Model

The test was done according to Molla and Kifle [8, 25], with a slight modification. Mice were fasted for 18 h, but with free access to water. The mice were grouped into 5 groups and preserved as described above. After one hour of treatment, 0.5 ml castor oil was given to each mouse. Then, 1 ml of 5% activated charcoal was administered by oral route. The mice were then sacrificed by cervical dislocation 2 h after castor oil administration. The small intestine was dissected out from pylorus to caecum and placed lengthwise on white paper. The total length of the intestine and the distance traveled by the charcoal meal were then measured and expressed as a peristaltic index:

(3) $$\begin{array}{c} \text{peristaltic index}\left(\text{PI}\right)=\frac{\text{distance travelled by charcoal meal}\times 100}{\text{whole length of small intestine}},\\ \text{percentage inhibition}=\frac{\text{PI of control group}-\text{PI of test group}\mathrm{\times 100}}{\text{PI of control group}}. \end{array}$$

### 2.8. Castor Oil-Induced Enteropooling Model

The method used by Suliman et al. [26] was used to evaluate the activity of the crude extract on castor oil-induced intestinal fluid accumulation. The mice were assembled and treated in the same way as described above. One hour later, 0.5 ml of castor oil was given to each mouse. One hour after the administration of castor oil, the mice were sacrificed by cervical dislocation and the small intestine of each mouse was weighed. Then, the intestinal content of all groups was transferred into a tube. After removal of the intestinal content, the intestine of each mouse was reweighed and the volume of each intestinal content was measured. Finally, the percent reductions in the volume and weight of intestinal content were determined using the following formulas:

(4) $$\begin{array}{c} \%\text{ reduction in weight of intestinal content}=\frac{\text{weight of intestinal content in NC}-\text{weight of intestinal content in TG}\times 100}{\text{weight of intestinal content in the negative group}}, \end{array}$$

where NC is the negative control group and TG is the treated group, and

(5) $$\begin{array}{c} \%\text{ reduction in volume of intestinal content}=\frac{\text{volume of intestinal content in NC}-\text{volume of intestinal content in TG}\times 100}{\text{volume of intestinal content in the negative group}}. \end{array}$$

### 2.9. *In Vivo* Antidiarrheal Index

The method used by Hussain et al. [27] was used to calculate the *in vivo* antidiarrheal index (ADI) for the test and control groups. The ADI was calculated using the following formula:

(6) $$\begin{array}{c} \text{ADI in vivo}=\sqrt[{3}]{\left(\text{Dfreq}\times \text{Gmeq}\times \text{Pfreq}\right)}, \end{array}$$

where Gmeq is the gut meal travel reduction as % of negative control, Pfreq is the reduction in the number of stools as % of the negative control, and Dfreq is the delay in defecation time or diarrhea onset which is calculated as follows:

(7) $$\begin{array}{c} \text{Dfreq}=\frac{\text{mean onset of diarrhea in the treated group}-\text{mean onset of diarrhea in the negative control}\times 100}{\text{mean onset of diarrhea in the negative control group}}. \end{array}$$

### 2.10. Statistical Analysis

The collected data were analyzed using the software Statistical Package for Social Sciences (SPSS), version 24. Thus, the results were expressed as mean ± standard error of means (SEM). The significance of differences between groups was analyzed by using one-way ANOVA followed by post hoc Tukey's test. A *P* value of less than 0.05 was considered statistically significant.

## 3. Results

### 3.1. The Percentage Yield of Extraction

The crude extract obtained at the end of the extraction process was 153 g (14.6%).

### 3.2. Acute Toxicity Test

With the acute toxicity test at the limit test dose of 2 g/kg, neither mortality nor changes related to behavioral, autonomic, neurologic, and physical profiles were observed within the first 24 h and during the 14-day follow-up. The dose was considered as the maximum tolerated dose, and thus, 1/5^th^, 1/10^th^, and 1/20^th^ doses of 2 g/kg were selected for the present study.

### 3.3. Effects of Crude Extract of *H. abyssinica* on Castor Oil-Induced Diarrheal Model

In the castor oil-induced diarrhea model, diarrhea was ostensible in all mice of the negative control group, for the next 4 h. The percentage inhibition of defecation by the crude extract was 42.85%, 55.71%, and 73.85% at the doses of 100 mg/kg, 200 mg/kg, and 400 mg/kg, respectively. Similarly, this was noticeably (87.5%) reduced by the oral administration of the standard drug (loperamide 3 mg/kg). All tested doses of the crude extract of *H. abyssinica* significantly (*P* < 0.001) delayed the onset of diarrhea when compared to the negative control. Likewise, all tested doses (100 mg/kg, 200 mg/kg, and 400 mg/kg) of the crude extract significantly (*P* < 0.05, *P* < 0.01, *P* < 0.001, respectively) reduced the number of wet feces and the total number of feces when compared to the negative control (Table 1).

### 3.4. Effects of Crude Extract of *H. abyssinica* on Castor Oil-Induced Intestinal Transit in Mice

The crude extract of *H. abyssinica* at the middle (200 mg/kg) and higher (400 mg/kg) doses significantly (*P* < 0.01 , *P* < 0.001, respectively) repressed propulsion of charcoal. However, the lower dose of the extract (100 mg/kg) lacks noticeable activity on the peristalsis of the intestine when compared to the negative control. As shown in Table 2, the percentage reduction of gastrointestinal transit of charcoal was 14.87%, 29.20%, and 54.00% for the crude extract at doses of 100 mg/kg, 200 mg/kg, and 400 mg/kg, respectively. Similarly, the standard drug (loperamide) reduced the small intestinal transit significantly (*P* < 0.001) with a percentage value of 67.1% (Table 2).

### 3.5. Effects of Crude Extract of *H. abyssinica* on Castor Oil-Induced Enteropooling

In the intestinal fluid accumulation model, the crude extract of *H. abyssinica* exhibited a significant reduction in both the average volume and weight of intestinal contents at the middle (200 mg/kg) and higher (400 mg/kg) doses of the extract as compared to the negative control. The percentage inhibition of weight of intestinal contents was 25% (*P* < 0.01) and 46% (*P* < 0.001) at doses of 200 mg/kg and 400 mg/kg, respectively, as compared to the negative control. Similarly, the percentage inhibition of volume of intestinal contents was 27.6% (*P* < 0.01) and 45.0% (*P* < 0.001) at doses of 200 mg/kg and 400 mg/kg, respectively, as compared to the negative control. Likewise, the percentage inhibition of volume and weight of intestinal contents by loperamide 3 mg/kg was 47.8% (*P* < 0.001) and 50.0% (*P* < 0.001), respectively, as compared to the negative control (Table 3).

### 3.6. Antidiarrheal Index

The *in vivo* ADI showed that the greatest ADI was achieved at the dose of 400 mg/kg of crude extract (81.24%) among the three doses of crude extracts. ADI increased with a dose for hydromethanolic crude extract of *H. abyssinica* but lower than the ADI of loperamide (99.48%). The crude extract of *H. abyssinica* displayed an ADI of 36.23%, 59.00%, and 81.24% at doses of 100 mg/kg, 200 mg/kg, and 400 mg/kg, respectively (Table 4).

## 4. Discussion

Plant-based medicines have been studied for the search of new antidiarrheal agents with advanced efficacy and safety. The activity of these medicinal plants on electrolyte secretion, water secretion, and GI transit was assessed by *in vivo* models [28]. Thus, the current study was aimed to evaluate the effectiveness and safety of *H. abyssinica* as an antidiarrheal agent. In the acute oral toxicity study, the crude extract of *H. abyssinica* at a single dose of 2 g/kg body weight orally did not cause any major toxicity and death. This result displayed that the LD_50_ of *H. abyssinica* extract is greater than 2 g/kg. Thus, this finding supports the study which presents the tough evidence of the nontoxic outcome of the medicinal plant [29].

The crude extract of *H. abyssinica* exhibited a dose-dependent antidiarrheal activity in all diarrheal parameters such as the total number of stools, weight of wet stools, onset of diarrhea, and number of wet stools when compared with the negative control group. This finding is consistent with previous similar studies [9, 14, 30]. Medicinal plants with antidiarrheal activity are known to delay the onset of diarrhea and decrease the consistency of fecal droppings and the number of wet stools as reported for *Saussurea lappa* [31], *Urena lobata* [32], *Lithocarpus dealbata,* and *Pterocarpus erinaceus* [33].

In the current study, a castor oil-induced diarrheal model was conducted to evaluate whether the crude extracts of *H. abyssinica* have an antidiarrheal effect or not. Then, other *in vivo* models such as antientropooling and antipropulsive were done in an attempt to suggest other possible mechanisms of action like antisecretory activity and decrease in GI transit activities by which they displayed antidiarrheal effect. In the enteropooling model, the higher dose (400 mg/kg) of the extract has a comparable antidiarrheal effect with loperamide 3 mg/kg. However, the lower dose (100 mg/kg) lacks a noticeable antidiarrheal effect which could be attributed to the inability of the phytoconstituents to reach sufficient concentration to achieve antisecretory effect and decrease in GI transit effects [34].

Castor oil was used in the induction of diarrhea through inflammatory and irritation activities on GI mucosa via ricinoleic acid. Lipases are responsible for the release of ricinoleic acid from castor oil [35]. Ricinoleic acid is known to produce different effects in the GIT such as inflammation of the gut, local irritation, and release of prostaglandin leading to fluid hypersecretion and intestinal hyperactivity that ultimately rises GI peristalsis, reduction in the activity of Na/K-ATPase in the intestine, and finally end up with diarrhea [36, 37]. Ricinoleic acid also forms salts with K^+^ and Na^+^ that will raise the permeability of the intestinal epithelium and constrain Na/K-ATPase, which in turn causes a cytotoxic consequence on intestinal cells [6]. Thus, the use of castor oil for the induction of diarrhea is sensible as it impressionists the pathophysiologic processes of motility and secretory diarrhea. In the present study, loperamide 3 mg/kg was used as the reference drug as it stimulates the absorption of glucose, electrolytes, and fluid through inhibition of prostaglandin activity [38].

In the castor oil-induced diarrheal model, the crude extract *H. abyssinica* revealed a significant reduction of the number of diarrheal episodes along with noticeable prolongation of the onset of the first diarrheal episode dose-dependently. This indicates that a relatively high dose of the crude extract is required to produce a significant antidiarrheal activity. This finding is in agreement with previous similar studies [9, 39]. The insignificant antidiarrheal effect of the crude extracts at the lower dose (100 mg/kg) could be because of the inability of the phytoconstituents to reach sufficient concentration to produce antidiarrheal effect. The crude extract of *H. abyssinica* produces antidiarrheal activity that may be accredited to, at least in part, its antimotility effect.

The crude extract reduced GI peristalsis in castor oil-treated mice as exhibited by a reduction in GI movement of the charcoal meal transit at the higher doses (400 mg/kg) of *H. abyssinica* crude extract. The reduction in GI peristalsis endorses intestinal electrolyte and water absorption. Similar results were reported with *Justicia schimperiana* antimotility effect for antidiarrheal activity [14].

The other likely mechanism of *H. abyssinica* for its antidiarrheal effect might be through antisecretory activity as it was apparent from the reduction in the total number of wet feces due to the attendance of phytoconstituents such as anthraquinones, phenols, tannins, terpenoids, saponins, glycosides, flavonoids, and alkaloids [8]; quercetin 3-O-*β*-glucoside, quercetin 3-O-*β*-glucuronide, quercetin glucuronide, and rutin [40]; protokosin, kosotoxin, *α*-kosin, kosidin, *β*-kosin, bitter acrid resin, tannic acid, and volatile oil [41, 42]; amino acids such as threonine, serine, aspartic acid, proline, glutamic acid, glycocol, valine, arginine alanine, methionine, cystine, leucine, tyrosine, iso-leucine, histidine, and phenylalanine [43]; minerals such as chromium, calcium, zinc, and other trace minerals [44]; and organic acids such as *α*-ketoglutaric acid, malic acid, fumaric acid, citric acid, glycolic acid, and succinic acid were identified [43]. The existence of tannates in medicinal plants produces effects like reduction in secretion and makes the GI mucosa more resistant [45, 46]. Tannins induce muscle relaxation through stimulation of the calcium pumping system or reduction of the intracellular Ca^2+^ inward current [47]. Tannins also denature proteins in the GI mucosa making protein tannates that make the GI mucosa more resistant and later decrease peristaltic movement. Tannins and tannic acid are water-soluble polyphenols that exist in many medicinal plants with antidiarrheal effects [48]. Flavonoids and tannins also rise the reabsorption of water and electrolytes by delaying castor oil-mediated NO production [39]. Diterpenes, sesquiterpenes, terpenes, terpenoids, and flavonoids are identified for hindering the release of prostaglandins and autacoids, thereby averting secretion and peristalsis induced by castor oil [49–51]. Steroids such as phytosterols and terpenoids such as abietic acid have been revealed to constrain the synthesis of prostaglandin E2, which are known to stimulate GI secretions [52, 53].

Moreover, the crude extract of *H. abyssinica* has shown anti-inflammatory activity like that of nonsteroidal anti-inflammatory drugs [54]. Nonsteroidal anti-inflammatory drugs could hinder ricinoleic acid-induced diarrhea apart from its inhibition on prostaglandin synthesis via their anti-inflammatory action [55]. Thus, it is rational to presume that the antidiarrheal effect of the crude extract of *H. abyssinica* could be accredited to anti-inflammatory effect. Most frequently used antidiarrheal medications act through averting the secretion of GI contents and/or reducing the GI peristalsis [25]. To evaluate the activity of medicinal plants on the GI peristalsis, an activated charcoal test is a chosen method [48, 56]. Activated charcoal was used in the GI motility test to serve as a marker. In the current study, the higher dose (400 mg/kg) of the crude extract repressed the charcoal marker which is similar to the reference drug (loperamide 3 mg/kg). This finding displayed that the crude extract possesses an antimotility effect which is followed by more absorption of water and electrolytes. This might be responsible for the reduction in the number of wet and total feces.

## 5. Conclusion

The crude extract of *H. abyssinica* showed antidiarrheal activity via inhibition of secretion and antimotility activity. Thus, the antidiarrheal effect of the leaves of *H. abyssinica* extract confirmed the traditional claim that the leaves of the plant are used for the treatment of diarrhea.

## Figures and Tables

**Table 1:** Effects of crude extract of *H. abyssinica* on castor oil-induced diarrhea.

Group	Onset of diarrhea (min)	Number of wet feces	Number of total feces	% inhibition of defecation
Control	58.50 ± 3.45	7.00 ± 0.73	9.83 ± 0.83	—
Crude 100 mg/kg	102.16 ± 7.79^a3b1^	4.00 ± 0.44^a1b1^	5.00 ± 0.68^a1b1^	42.85
Crude 200 mg/kg	132.33 ± 6.33^a3^	3.10 ± 0.22^a2b2^	3.72 ± 0.53^a2^	55.71
Crude 400 mg/kg	137.16 ± 7.84^a3^	1.83 ± 0.21^a3^	2.66 ± 0.33^a3^	73.85
Loperamide 3 mg/kg	169.33 ± 6.075^a3^	1 ± 0.25^a3^	2.0 ± 0.21^a3^	87.50

Data are expressed as mean ± SEM (*n* = 6). ^a^Compared to negative control. ^b^Compared to loperamide 3 mg/kg. ^1^*P* < 0.05, ^2^*P* < 0.01, and ^3^*P* < 0.001. Negative controls received 10 ml/kg distilled water.

**Table 2:** Effects of crude extract of *H. abyssinica* on castor oil-induced gastrointestinal motility.

Dose administered	Length of small intestine (cm)	Distance moved by the charcoal meal (cm)	Peristaltic index (%)	% inhibition
Control	57.10 ± 1.24	49.50 ± 1.43	86.70 ± 1.48	—
100 mg/kg	58.60 ± 0.95	43.26 ± 3.73^b3^	73.80 ± 4.91^b3^	14.87
200 mg/kg	59.00 ± 1.12	36.20 ± 1.45^a2b3^	61.35 ± 2.88^a2b3^	29.20
400 mg/kg	57.16 ± 0.93	22.00 ± 1.32^a3^	39.85 ± 1.97^a3^	54.00
Loperamide 3 mg/kg	55.5 ± 1.51	15.83 ± 0.94^a3^	28.52 ± 1.08^a3^	67.10

Data are expressed as mean ± SEM (*n* = 6). ^a^Compared to negative control. ^b^Compared to loperamide 3 mg/kg. ^1^*P* < 0.05, ^2^*P* < 0.01, and ^3^*P* < 0.001. Negative controls received 10 ml/kg distilled water.

**Table 3:** Effects of crude extract of *H. abyssinica* on castor oil-induced enteropooling

Dose administered	Mean volume of small intestinal content (g)	% inhibition	Mean weight of small intestinal content (ml)	% inhibition
Control	0.69 ± 0.09	—	0.76 ± 0.01	—
100 mg/kg	0.66 ± 0.04^b3^	4.30	0.70 ± 0.02	7.89
200 mg/kg	0.50 ± 0.02^a2b2^	27.60	0.57 ± 0.06^a2^	25.00
400 mg/kg	0.38 ± 0.02^a3^	45.00	0.41 ± 0.02^a3^	46.00
Loperamide 3 mg/kg	0.36 ± 0.02^a3^	47.80	0.38 ± 0.02^a3^	50.00

Data are expressed as mean ± SEM (*n* = 6). ^a^Compared to negative control. ^b^Compared to loperamide 3 mg/kg. ^1^*P* < 0.05, ^2^*P* < 0.01, and ^3^*P* < 0.001. Negative controls received 10 ml/kg distilled water.

**Table 4:** *In vivo* antidiarrheal index of crude extract of *H. abyssinica*.

Dose administered	Delay in defecation time (Dfreq) (%)	Gut meal travel distance (Gmeq) (%)	Purging frequency in number of wet stools (%)	*In vivo*ADI
Control	—	—	—	—
100 mg/kg	74.62	14.87	42.85	36.23
200 mg/kg	126.20	29.20	55.71	59.00
400 mg/kg	134.46	54.00	73.85	81.24
Loperamide 3 mg/kg	189.45	67.10	77.50	99.48

ADI = antidiarrheal index.

## Data Availability

The datasets used and/or analyzed during the current study are available from the corresponding author upon reasonable request.
